# Internalized Weight Stigma: Prevalence and Association With Psychiatric Disorder Among Overweight and Obese Individuals

**DOI:** 10.7759/cureus.18577

**Published:** 2021-10-07

**Authors:** Bandar F Almutairi, Khaled W Alsaygh, Mazen M Altamimi, Abdulrahman S Alshammari, Ahmed M Alsomali, Sultan M Alanazi, Saud M Alzahrani, Ameer S Alsaad, Georgios Zacharakis

**Affiliations:** 1 Psychiatry, Prince Sattam Bin Abdulaziz University, Al Kharj, SAU; 2 Internal Medicine, Prince Sattam Bin Abdulaziz University, Al Kharj, SAU

**Keywords:** internalized weight stigma, prevalence, depression, anxiety, obese, overweight

## Abstract

Background and objective

Unlike weight stigma, internalized weight stigma (IWS) may be a common but still underreported problem. With the recent emergence of studies investigating its various aspects in Arab-speaking countries, there is still scant data on its incidence and severity in the literature. In light of this, the aim of this study was to evaluate the prevalence of IWS and its association with psychiatric disorders and sociodemographic factors among overweight and obese individuals in a sample from a Saudi population.

Methods

A cross-sectional study was conducted among a Saudi population using a convenience sample technique and 868 individuals were enrolled to participate in this study. They were asked to fill out an electronic questionnaire about IWS, demographics, and other parameters such as Patient Health Questionnaire (PHQ)-9 and General Anxiety Disorder (GAD)-7. The association was tested using an independent t-test and chi-square test.

Results

The overall prevalence of internalized stigma in this study was 57%. Higher levels of internalization were more prevalent among younger respondents. We found that females were more prone to internalize weight stigma, at a slightly higher rate than males (59.26% vs. 53.66%), but this difference was not statistically significant. The other sociodemographic factors associated with higher internalization were as follows: being widowed, married, retired, or housewife. Those with higher IWS levels were individuals with higher BMI and with previous experiences of weight stigma. In addition, higher internalization was associated with the development of severe depression and anxiety (p<0.001).

Conclusion

The prevalence of IWS among overweight and obese individuals was found to be high, and it is highly associated with the development of severe depression and anxiety. There is a need to raise awareness about obesity stigma to help tackle IWS in overweight and obese individuals and to promote their quality of life.

## Introduction

Stigma is defined as negative views or stereotypes assigned to a person or people when their characteristics are considered to be different from or inferior to societal norms [1]. This is a multidimensional concept. Enacted stigma refers to the discrimination that is experienced directly in areas of social life, such as discrimination in social relationships, employment, reduced access to public services, and housing. Internalized or self‐stigma is the type of stigma characterized by self‐devaluation and the fear of enacted stigma resulting from one's identification with a stigmatized group [2]. Stigmatization has been associated with many aspects, including race, gender, socioeconomic status, age, and chronic health conditions such as mental illnesses, epilepsy, AIDS/HIV, tuberculosis, and leprosy [3].

Obese and overweight individuals are also prone to stigmatization and discrimination, besides being susceptible to serious complications including hypertension, dyslipidemia, diabetes mellitus, stroke, and coronary heart disease [4]. They suffer from weight stigma in their life, which comprises the negative attitudes, assumptions, and judgments that are directed toward them for being obese. They are perceived by individuals and society as being lazy and sloppy people, lacking the willpower and self‐discipline, and as unmotivated and noncompliant with societal norms [5]; consequently, they face prejudice and discrimination in the fields of education, employment, and healthcare [6], which in turn induces physiological stress, eating disorders, lower physical activity, increased food consumption, and thereby make them more vulnerable to psychological distress such as depression, anxiety, low self-esteem, and body image dissatisfaction [7]. Internalized weight stigma (IWS) is a worrisome condition, as it is evident that its psychological and mental outcomes are worse than those related to weight stigma alone [8]. It has significantly contributed to poor psychosocial and emotional outcomes, including low self-esteem, depression, anxiety, body image concern, and binge eating [9,10]. The prevalence of weight stigma has increased enormously in the past decade and is now comparable to racial discrimination in America [11]. In addition, 44% of US adults experience IWS [12]. A recent study conducted among the Saudi Arabian population estimated the prevalence of weight stigma to be 81.55% [13]. IWS has received little attention compared to the weight stigma that is expressed by others toward obese people, and to the best of our knowledge, it has never been investigated in Arabic-speaking countries. Therefore, in this study, we investigated its prevalence and association with sociodemographic characteristics and psychiatric disorders in a sample of obese and overweight individuals among the Saudi population.

## Materials and methods

Study design

This was a population-based cross-sectional study and was conducted between February and May of 2021.

Study setting and participants

A total of 886 participants were included in this study. Inclusion criteria were obese and overweight individuals aged 18 years or more who were willing to participate in the survey. Exclusion criteria were those who were younger than 18 years, and individuals with normal weight, or those who were underweight. The convenience sample technique was employed for the collection of study data using a self-administered electronic questionnaire written in Arabic, which was distributed to the target Saudi population through social media platforms.

Measures

The self-administered electronic questionnaire consisted of six domains: sociodemographic information, anthropometric measurements, history of experienced weight stigma, internalization of weight stigma, and assessment of psychiatric disorders (depression and anxiety). These measures are described below. 

Sociodemographic Information

Respondents answered questions regarding their age, gender, level of education, marital status, work status, and monthly income.

Anthropometric Measurements

Participants self-reported their weight and height [BMI was calculated as weight (kg) divided by height (m^2^)]. Participants were classified into four categories based on their BMI - overweight: those with a BMI greater than or equal to 25-29.9 kg/m^2^; obesity class one: those with a BMI of 30-34.9 kg/m^2^; obesity class two: those with a BMI of 35-39.9 kg/m^2^; and obesity class three: those with a BMI greater than or equal to 40 kg/m^2^.

Weight Stigma

The participants were asked three (yes/no) questions about whether they had ever experienced discrimination, teasing, or unfair treatment due to their weight [14].

Internalized Weight Stigma Measurement

IWS was measured using the Weight Self-Stigma Questionnaire (WSSQ), which entails a Likert scale composed of 12 items that are divided into two subscales that measure weight-related self-devaluation and fear of enacted stigma. Items one to six constitute the self-evaluation subscale, and items seven to 12 constitute the fear of the enacted stigma subscale. Each item was rated on a scale of 1 (completely disagree) to 5 (completely agree). The lowest score is 12, while the highest is 60; the higher the score, the higher the sense of self-stigma. A valid and reliable Arabic version was used in this study [15]. No cut-offs have been established for WSSQ scores. We used the mean as a cut-off point; the mean in this study was 40.93 ± 9.00, and we considered those who were above the mean to have a high level of IWS.

Psychiatric Disorders Assessment

To assess the presence and measure the severity of depression and anxiety, the Patient Health Questionnaire (PHQ)-9 and General Anxiety Disorder (GAD)-7 were used.

PHQ-9: this questionnaire was used to assess depression symptoms. It is a nine-item depression screening instrument, based on the Diagnostic and Statistical Manual of Mental Disorders, fourth edition (DSM-IV). It asks responders to answer a question on how often they have been bothered by each of the PHQ-9 symptoms over the last two weeks. Each answer was scored from 0 (not at all) to 3 (nearly every day), providing a 0-27 severity score [16]. A validated Arabic version of the questionnaire was used in this study.

GAD-7: anxiety was assessed using the GAD-7, which is a seven-item instrument based on the Diagnostic and Statistical Manual of Mental Disorders, fifth edition (DSM-V). It asks responders to rate the frequency of anxiety symptoms in the last two weeks. With a score assigned to each answerer from 0 (not at all) to 3 (nearly every day), providing a 0-21 severity score [17]. A validated Arabic version of the questionnaire was used in this study.

Statistical analysis

Data were collected using Google Form (Google LLC, Mountain View, CA) and then converted into Microsoft Excel (Microsoft Corp, Redmond, WA) and analyzed using SPSS Statistics version 25.0 (IBM, Armonk, NY). Numerical variables were presented as mean and standard deviation, while categorical variables were presented as frequencies and percentages. An independent t-test was used for comparisons between high internalization and low internalization groups with respect to all numerical variables, while the chi-square test was used for all categorical variables. Statistical significance was set at a p-value of <0.05.

Ethical approval

The study was approved by the Ethics Committee at Prince Sattam Bin Abdulaziz University (REC-HSD-71-2021).

## Results

Sample characteristics

A total of 886 respondents completed the questionnaire. Table 1 shows the baseline characteristics of the study population. The average age of the participants was 30.87 ± 9.40 years. The sample comprised 38.48% (n = 341) males and 61.52% (n = 545) females. Regarding marital status at baseline, 50.11% (n = 444) were single, 42.43% (n = 376) were married, 5.41% (n = 48) were divorced, and 1.58% (n = 14) were widowed. The most common educational degree was a bachelor’s degree, accounting for 62.97% (n = 558). Regarding occupation, 27.99% (n = 248) were students, 28.89% (n = 256) were employees, 18.51% (n = 164) were housewives, 21.67% (n = 192) were unemployed, 1.80% (n = 16) were freelancers, and 0.67% (n = 6) were retired. Approximately 72.00% (n = 638) of the participants had a monthly income of SAR 5,000 or less. Overweight participants comprised 53.31% (n = 474), and those with obesity class one were 27.89% (n = 248), class two accounted for 12.14% (n = 108), and class three 6.29% (n = 56).

The mean WSSQ score was 40.93 ± 9.00, and at least 57% endorsed the mean levels of WSSQ. When asked whether they had ever experienced discrimination because of their weight, 42.43% (n = 376) answered yes, and when asked whether they had ever been treated unfairly because of their weight, 30.69% (n = 272) answered yes, and lastly, when asked whether they had ever been teased because of their weight, 55.75% (n = 494) responded by answering yes (Table 1).

Regarding PHQ-9, 222 were classified as having minimal depression, accounting for 24.97%, those with mild depression accounted for 35.77% (n = 318), 20.02% (n = 178) were classified as having moderate depression, 11.92% (n = 106) had moderately severe depression, and 6.97% (n = 62) had severe depression. The classification of patients based on scores on the GAD-7 scale were as follows - minimal anxiety: 30.82% (n = 274); mild anxiety: 37.34% (n = 332); moderate anxiety: 15.29% (n = 136); and severe anxiety: 16.19% (n = 144) (Table 1).

**Table 1:** Baseline characteristics of the study population SD: standard deviation; SAR: Saudi riyal; BMI: body mass index; WSSQ: Weight Self-Stigma Questionnaire

Variables	Values	
Age (years), mean ± SD	30.87 ± 9.40	
Gender, n (%)	Male	341 (38.48%)
	Female	545 (61.52%)
Marital status, n (%)	Single	448 (50.56%)
	Married	376 (42.43%)
	Divorced	48 (5.41%)
	Widowed	14 (1.58%)
Education, n (%)	High school or less	284 (32.05%)
	Bachelor degree	558 (62.97%)
	Post-graduate	44 (4.96%)
Occupation, n (%)	Student	248 (27.99%)
	Employee	256 (28.89%)
	Housewife	164 (18.51%)
	Unemployed	192 (21.67%)
	Freelancer	16 (1.80%)
	Retired	10 (1.12%)
Monthly income (SAR), n (%)	≤5,000	638 (72.00%)
	5,000–9,999	108 (12.18%)
	10,000–19,999	100 (11.28%)
	20,000–29,999	18 (2.03%)
	≥30,000	22 (2.48%)
BMI category, n (%)	Overweight	474 (53.49%)
	Obesity class one	248 (27.99%)
	Obesity class two	108 (12.19%)
	Obesity class three	56 (6.32%)
Have you ever been treated unfairly because of your weight? n (%)	Yes	272 (30.69%)
	No	614 (69.30%)
Have you ever been teased because of your weight? n (%)	Yes	494 (55.75%)
	No	392 (44.24%)
Have you ever been discriminated against because of your weight? n (%)	Yes	376 (42.43%)
	No	510 (57.56%)
Internalized weight stigma (WSSQ score), mean ± SD	40.93 ± 9.00	
Depression classification, n (%)	Minimal depression	222 (25.05%)
	Mild depression	318 (35.89%)
	Moderate depression	178 (20.09%)
	Moderately severe depression	106 (11.96%)
	Severe depression	62 (6.99%)
Anxiety classification, n (%)	Minimal anxiety	274 (30.92%)
	Mild anxiety	332 (37.47%)
	Moderate anxiety	136 (15.35%)
	Severe anxiety	144 (16.25%)

Association of sociodemographic characteristics with WSSQ score

Table 2 shows the association between higher internalization levels and various sociodemographic characteristics. The mean age of people with higher internalization was 29.38 ± 8.90 years, which was associated with a higher WSSQ score (p<0.001). A total of 183 (53.66%) and 323 (59.26%) males and females had higher internalization, respectively, although this association was not statistically significant. Marital status was significantly associated with higher internalization (p<0.001), with 49.10% (n = 220) being single, 67.02% (n = 252) were married, 45.83% (n = 22) were divorced, and 85.71% (n = 12) were widowed. The prevalence of higher internalization based on types of occupations was as follows: 47.85% (n = 118) students; 58.59% (n = 150) employees; 68.29% (n = 112) housewives; 57.29% (n = 110) unemployed; 50.00% (n = 8) freelancers; and 70.00% (n = 7) retired (p<0.001). BMI category was also found to be significantly associated with higher internalization. The results showed that 254 (53.58%) among overweight, 140 (56.45%) among obese class one, 76 (70.37%) among obesity class two, and 36 (64.28%) among class three had high levels of internalization (p<0.001). On the other hand, education levels and monthly income were statistically insignificant factors, and those with SAR 5,000-9,999 monthly income accounted for 70.00% (n = 70) among the high internalization group.

**Table 2:** Association of sociodemographic characteristics with WSSQ score SD: standard deviation; SAR: Saudi riyal; BMI: body mass index; WSSQ: Weight Self-Stigma Questionnaire

Variables	Internalized weight stigma			P-value
		High internalization (n = 506)	Low internalization (n = 380)	
Age (years), mean ± SD		29.38 ± 8.90	31.99 ± 9.61	<0.001
Gender, n (%)	Male	183 (53.66%)	158 (46.33%)	0.101
	Female	323 (59.26%)	222 (40.73%)	
Education, n (%)	High school or less	168 (59.15%)	116 (40.84%)	0.683
	Bachelor degree	314 (56.27%)	244 (43.72%)	
	Post-graduate	24 (54.54%)	20 (45.45%)	
Marital status, n (%)	Single	220 (49.10%)	228 (50.89%)	<0.001
	Married	252 (67.02%)	124 (32.97%)	
	Divorced	22 (45.80%)	26 (54.16%)	
	Widowed	12 (85.71%)	2 (14.28%)	
Occupation, n (%)	Student	118 (47.58%)	130 (52.41%)	<0.001
	Employee	150 (58.59%)	106 (41.40%)	
	Housewife	112 (68.29%)	52 (31.70%)	
	Unemployed	110 (57.29%)	82 (42.70%)	
	Freelancer	8 (50.00%)	8 (50.00%)	
	Retired	7 (70.00%)	3 (30.00%)	
Monthly income (SAR), n (%)	≤5,000	352 (55.17%)	286 (44.82%)	0.072
	5,000–9,999	70 (70.00%)	30 (30.00%)	
	10,000–19,999	60 (55.55%)	48 (44.44%)	
	20,000–29,999	12 (66.66%)	6 (33.33%)	
	≥30,000	12 (54.54%)	10 (45.45%)	
BMI category, n (%)	Overweight	254 (53.58%)	220 (46.41%)	0.01
	Obesity class one	140 (56.45%)	108 (43.54%)	
	Obesity class two	76 (70.37%)	32 (29.63%)	
	Obesity class three	36 (64.28%)	20 (35.71%)	

Association of stigma experience and psychiatric disorders with WSSQ score

Table 3 shows the association between higher internalization and stigma experience as well as psychiatric disorders. Those who had been treated unfairly, teased, or discriminated against because of their weight were more likely to develop a higher internalization of weight stigma (p<0.001). Higher internalization was found to be influential in depression and anxiety development in 74.19% (n = 46) of patients with severe depression, and 70.83% (n = 102) with severe anxiety tended to have a higher internalization (p<0.001).

**Table 3:** Association of weight stigma experience and psychiatric disorders with WSSQ score WSSQ: Weight Self-Stigma Questionnaire

Variables	Internalized weight stigma			P-value
	N (%)			
Have you ever been treated unfairly because of your weight?	Yes	208 (76.47%)	64 (23.52%)	<0.001
	No	298 (48.53%)	316 (51.46%)	
Have you ever been teased because of your weight?	Yes	338 (68.42%)	156 (31.57%)	<0.001
	No	168 (42.85%)	224 (57.14%)	
Have you ever been discriminated against because of your weight?	Yes	262 (69.68%)	114 (30.31%)	<0.001
	No	244 (47.84%)	266 (52.15%)	
Depression category	Minimal depression	102 (45.94%)	120 (54.05%)	<0.001
	Mild depression	184 (57.86%)	134 (42.13%)	
	Moderate depression	116 (65.16%)	62 (34.83%)	
	Moderately severe depression	58 (54.71%)	48 (45.28%)	
	Severe depression	46 (74.19%)	16 (25.80%)	
Anxiety category	Minimal anxiety	118 (43.06%)	156 (56.93%)	<0.001
	Mild anxiety	194 (58.43%)	138 (41.56%)	
	Moderate anxiety	92 (67.64%)	44 (32.35%)	
	Severe anxiety	102 (70.83%)	42 (29.16%)	

## Discussion

IWS might be a common yet still underreported problem with some studies finding that it is linked to many psychological problems. These findings are worrisome as the consequence of such a correlation might be devastating. In Saudi Arabia, it has never been investigated before, and data regarding its prevalence are scarce. In this study, we aimed to investigate the prevalence of IWS and examine its association with sociodemographic characteristics and psychiatric disorders.

According to our findings, the prevalence of individuals who reported a high level of IWS was 57%. A study of a sample of adults in a commercial weight management program showed a similar result of 60% [18]. Other studies have shown lower rates, such as those reported among US adults, which reach up to 44% [12], and from among a sample of patients with type II diabetes (45.6%) [19]. The rate of IWS in our study is relatively high, although it is sensible to expect such a high rate as another study has shown that the rate of weight stigma is high in Saudi Arabia [13]. As a consequence, people who are obese or overweight will probably encounter more stigmatization and discrimination in their daily life. Our findings affirm this observation; when asked whether they had encountered discrimination, teasing, or had been treated unfairly because of their weight, a high portion of the sample responded positively by saying yes at a rate of 42.43%, 55.75%, and 30.69%, respectively; moreover, these results have shown to be significantly associated with such people developing a high level of IWS (p<0.001), and previous studies have also found that those who experienced any form of weight stigmatization end up having a high level of IWS [12,18].

We determined the association between having a high level of IWS and sociodemographic characteristics. With regard to gender, previous studies have found that females are more vulnerable to higher levels of internalization relative to men [20,21]. However, in our study, no association between gender and IWS was observed. Concerning age, our results have shown a significant association, with a lower mean age for those with a high level of internalization (p<0.001), which is consistent with the results of previous studies, where younger individuals were found to be more vulnerable to developing higher internalization [12,22]. Interestingly, married and widowed participants endorsed higher levels of IWS compared to single and divorced respondents (p<0.001). In contrast, one study found that being married or widowed was associated with a lower level of internalization [18]. We found that being a housewife or retired was associated with a high level of internalization (p<0.001), while those who were students and employees tended to have lower levels of IWS. We believe that being a housewife or retired makes individuals less active in comparison to being a student or employee, which may lead them to be more prone to developing obesity and being more susceptible to IWS, as it was found that obesity is more prevalent among housewives than employed women [23]. With regard to BMI, previous evidence has been inconsistent. Several studies have observed a significant association between IWS and BMI [24-26], whereas other studies have found no significant association [27,28]. In our sample, BMI was significantly associated with high internalization, especially among individuals belonging to obesity class two or higher (p<0.001).

This study also explored the association between internalized stigma and psychiatric disorders such as depression and anxiety. As expected, we found that internalized stigma was significantly associated with psychiatric disorders (p<0.001). Severe depression and anxiety are highly prevalent among those who reported higher internalization, which is in line with previous research showing higher rates of depression and anxiety in overweight and obese individuals who internalize weight-biased attitudes [9,29,30].

Our findings should be interpreted in light of several limitations and strengths. First, as with all cross-sectional survey research, this study design does not establish causality and we cannot rule out the possibility of response bias. Secondly, this study used the tool WSSQ, and presumably, using a different questionnaire or criteria for IWS might lead to a different outcome. A key strength of this research lies in the fact that it was the first local study to examine the prevalence and association between IWS and psychiatric disorders among the general population and it involved a relatively large sample size.

## Conclusions

The prevalence of IWS among overweight and obese individuals tested in this study was high. This problem is more prevalent among younger people and is highly associated with marital status, occupation, higher BMI, and with those who encounter any type of stigma; it has also been shown to be significantly associated with depression and anxiety. A comprehensive approach is needed to promote a better quality of life and tackle IWS in overweight and obese individuals. This research has shed some light on this problem, and we call for implementing adequate measures to raise awareness about obesity stigma among the population to decrease its effect on overweight and obese individuals.
